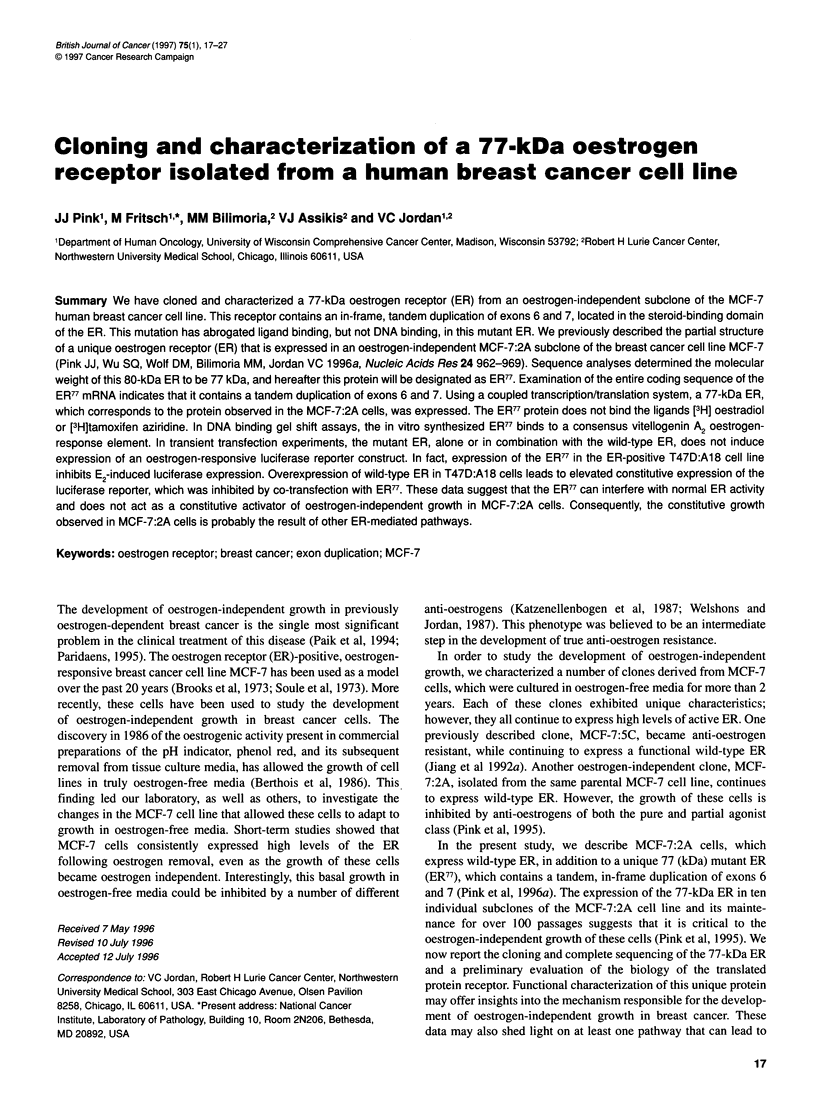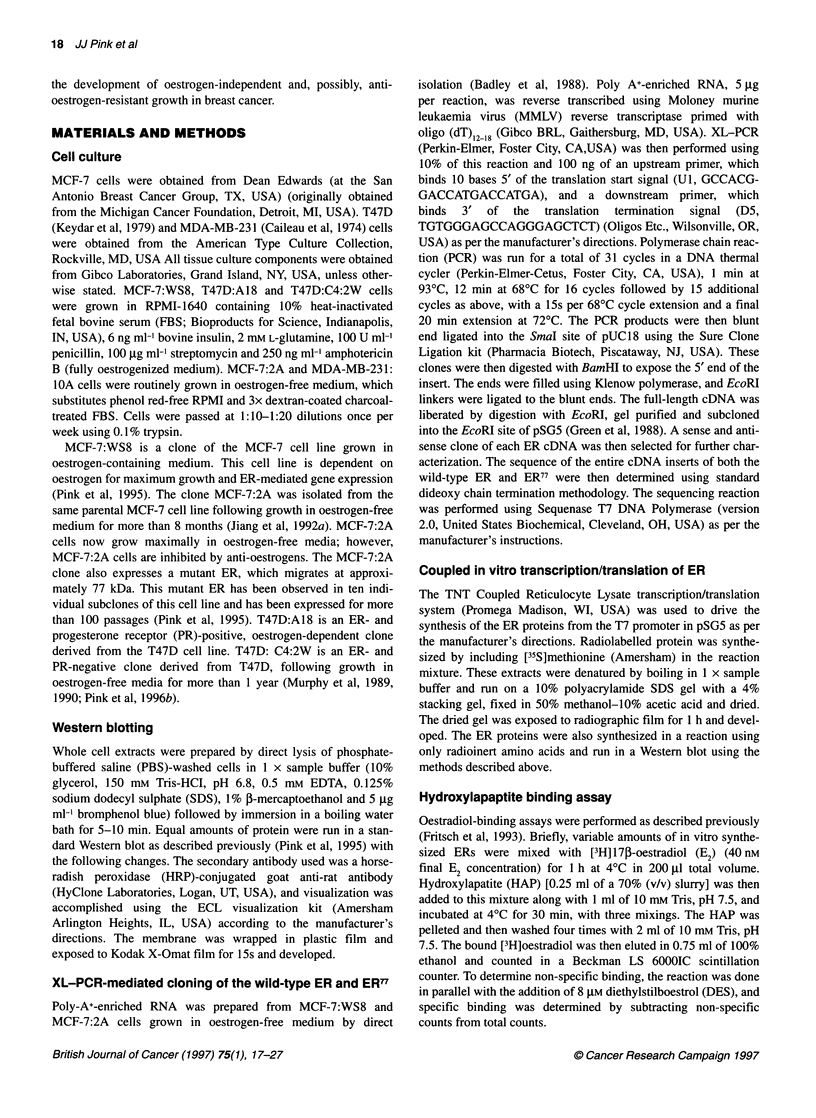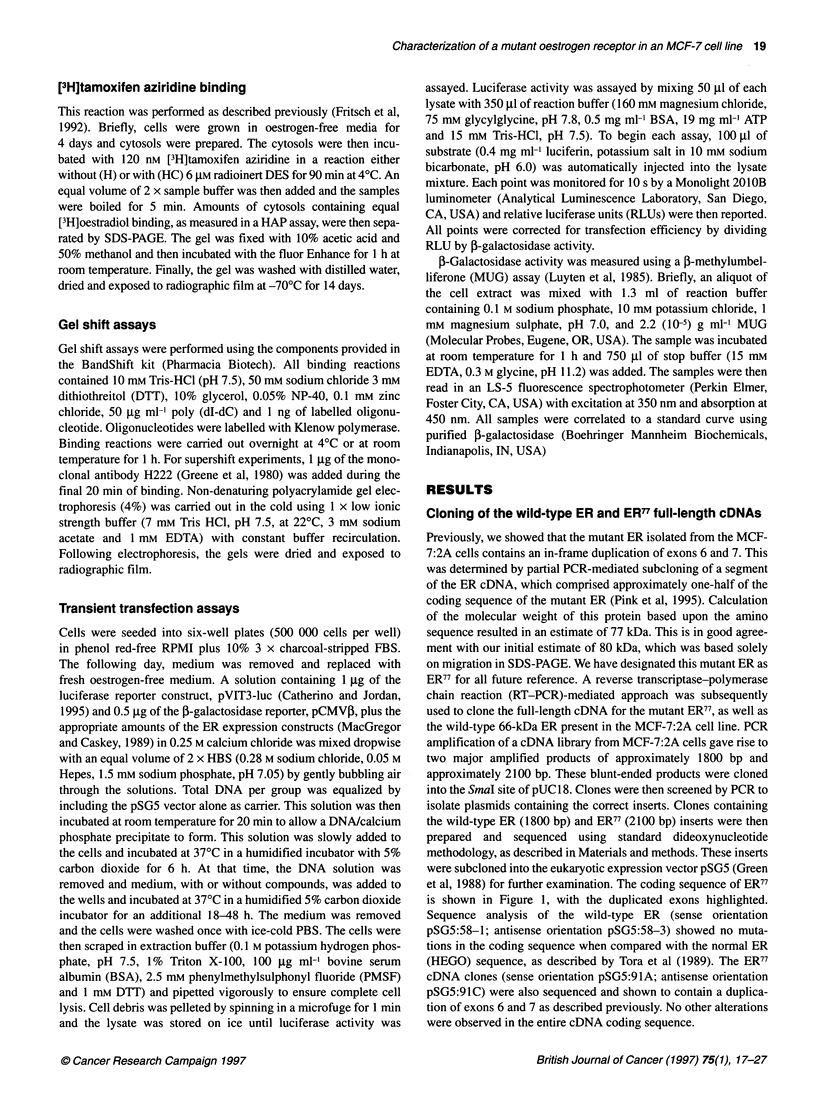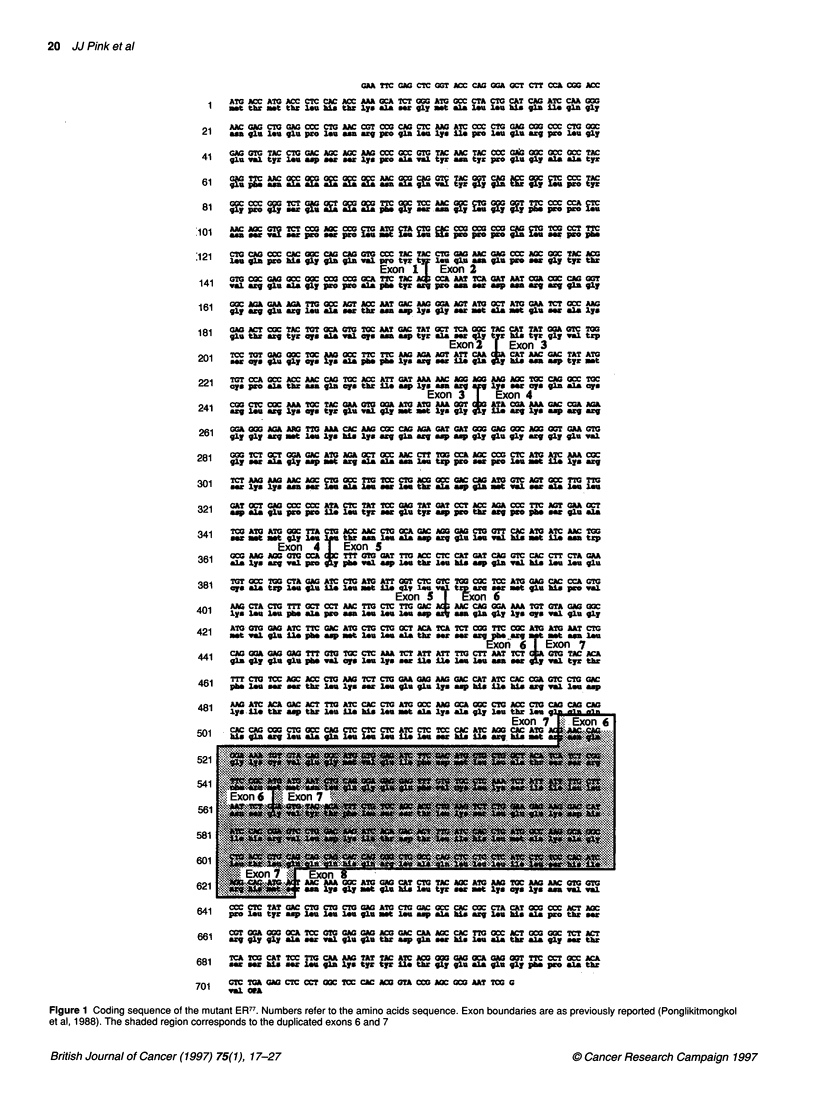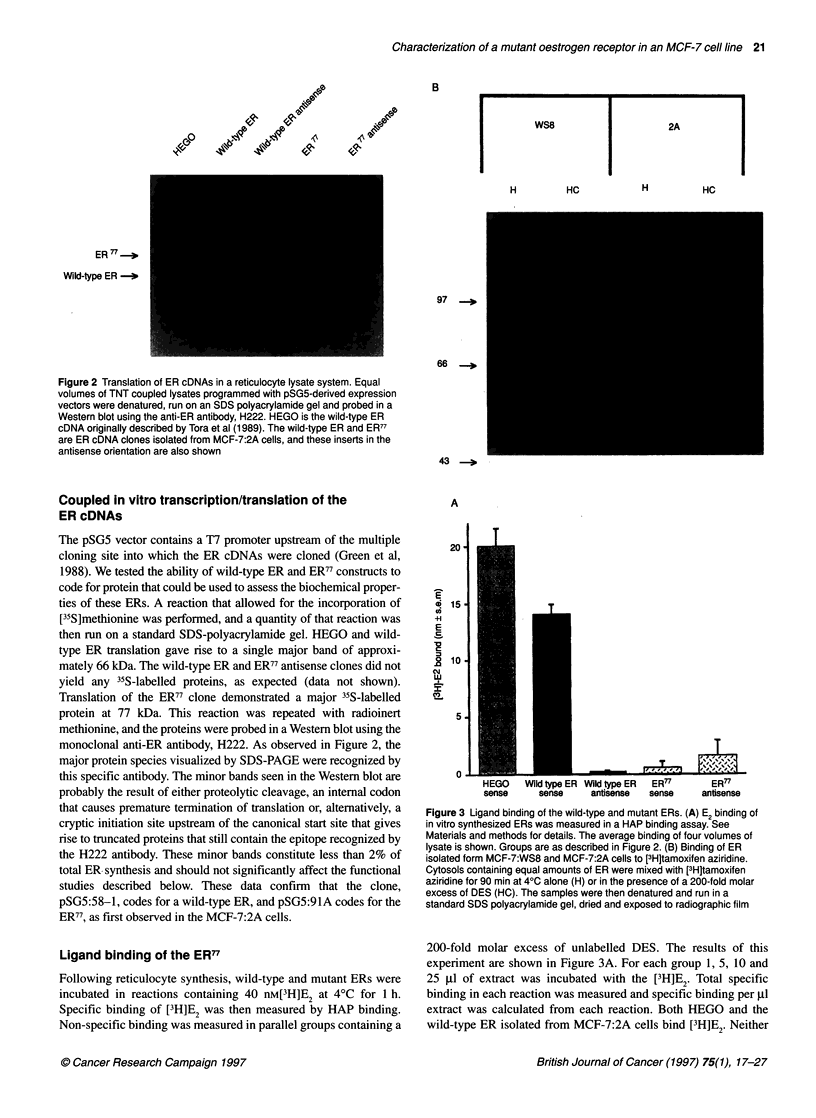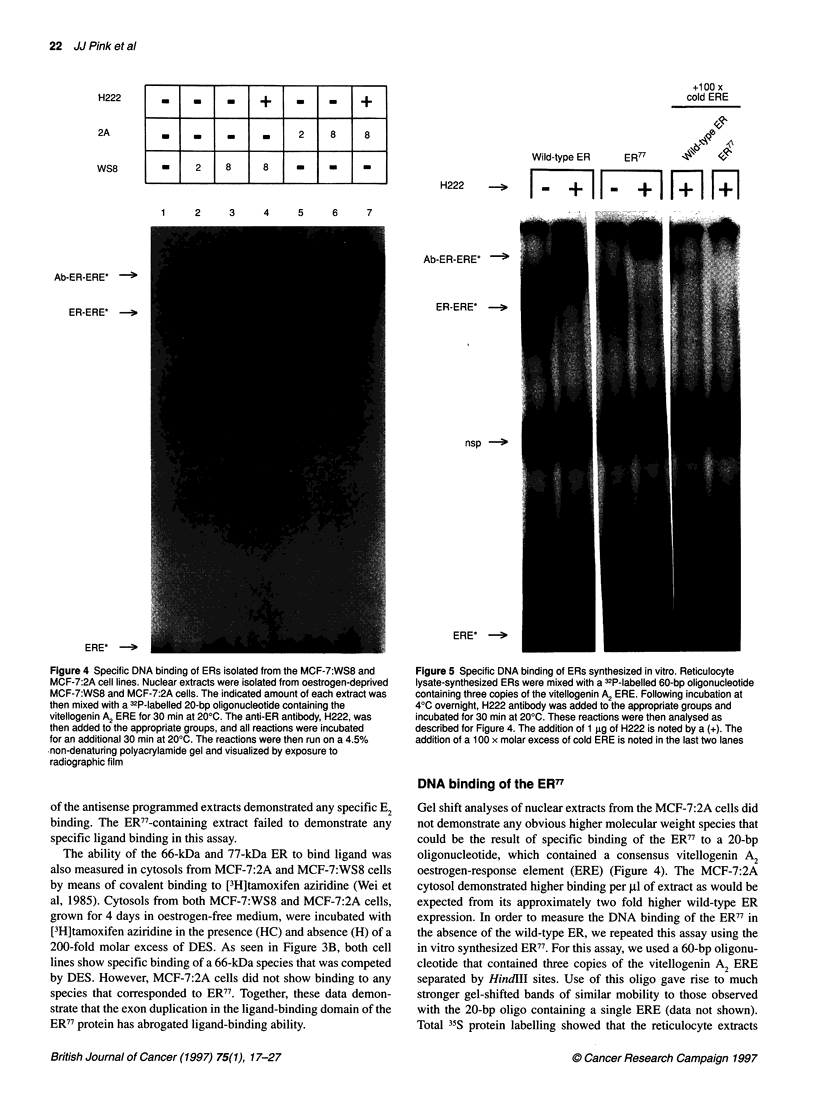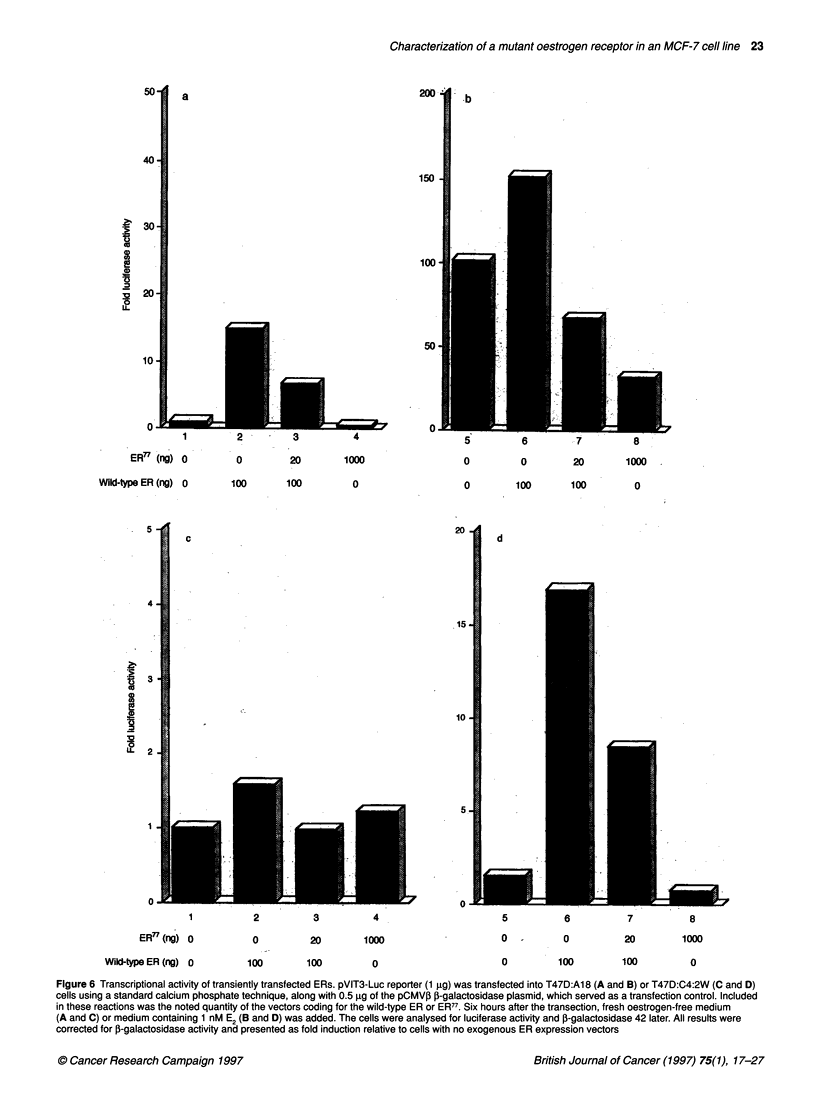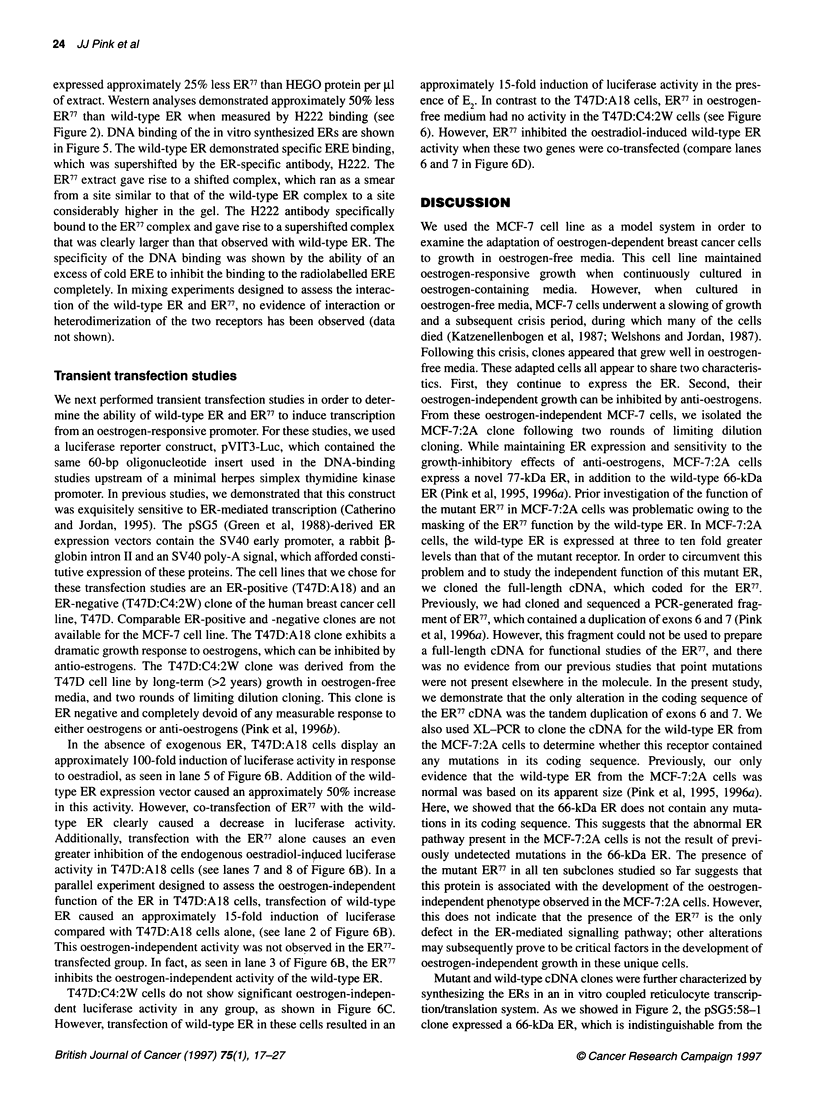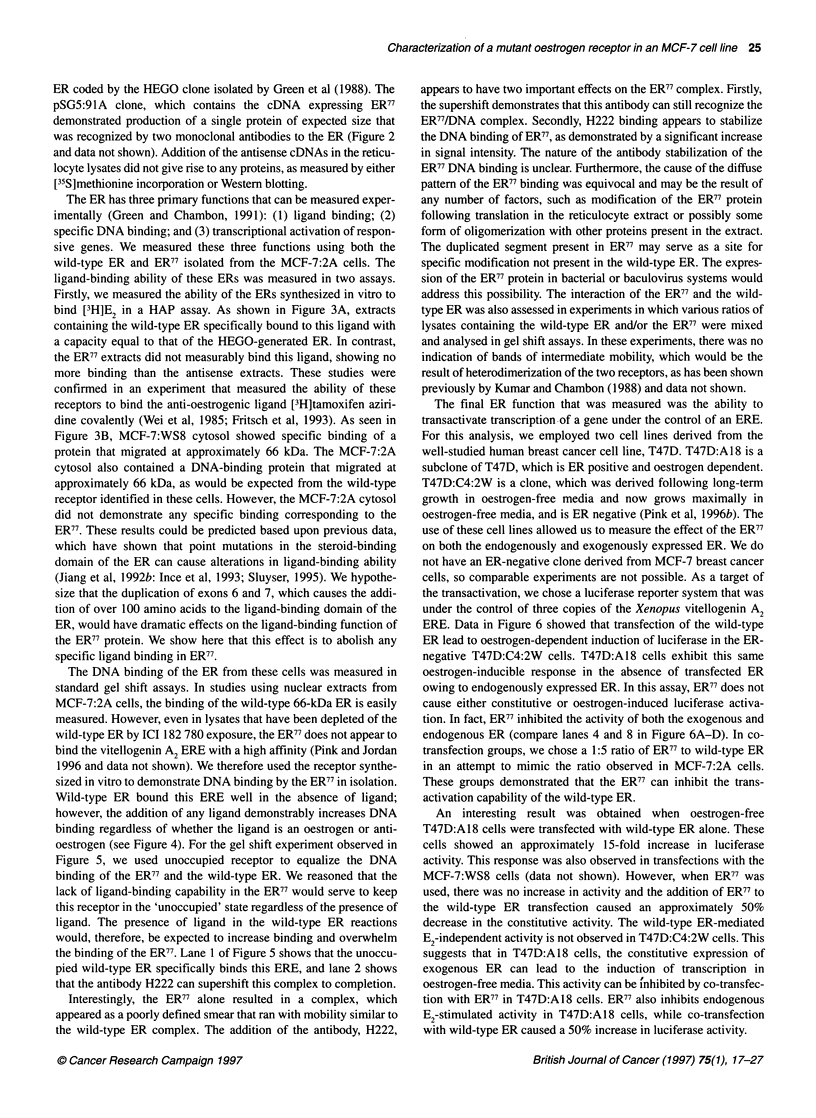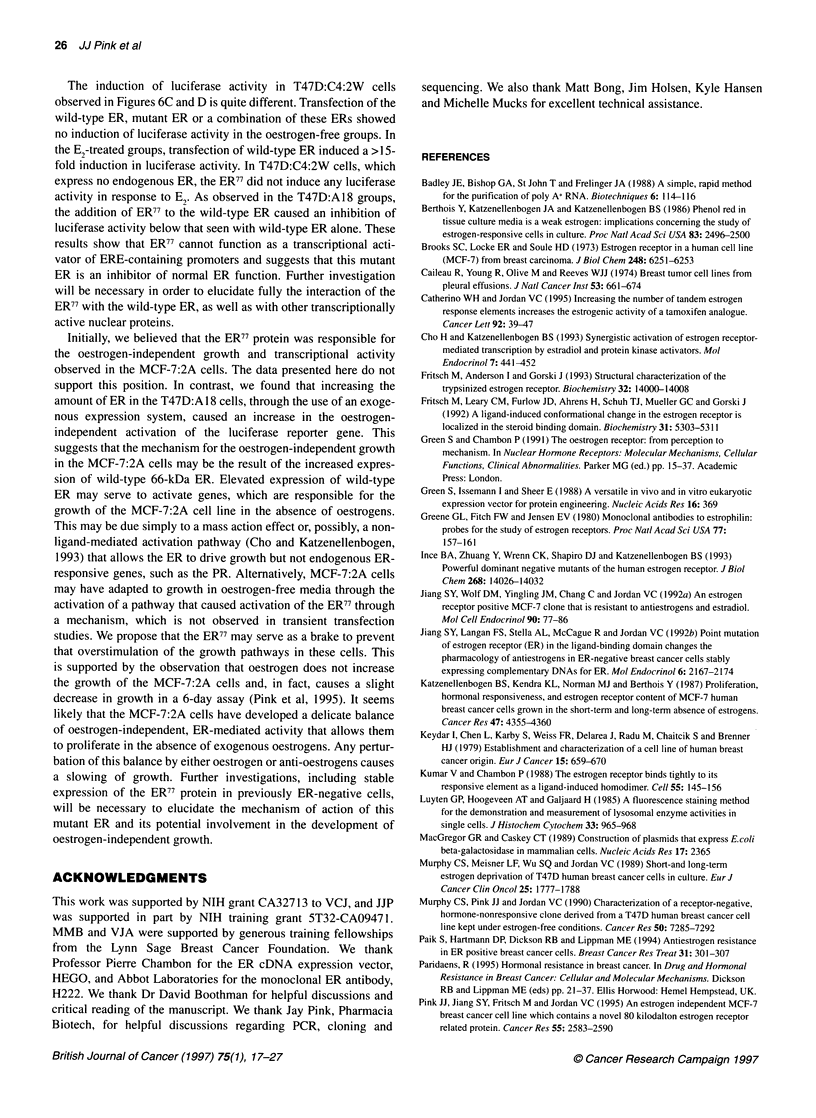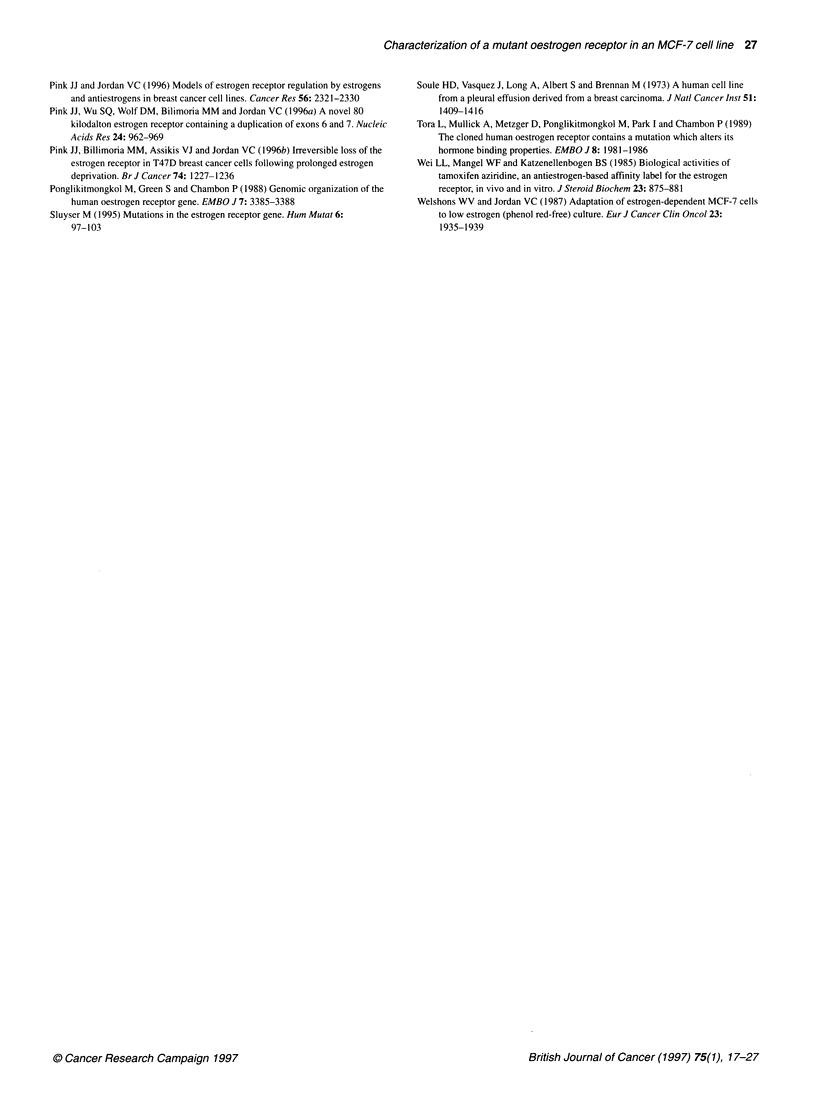# Cloning and characterization of a 77-kDa oestrogen receptor isolated from a human breast cancer cell line.

**DOI:** 10.1038/bjc.1997.4

**Published:** 1997

**Authors:** J. J. Pink, M. Fritsch, M. M. Bilimoria, V. J. Assikis, V. C. Jordan

**Affiliations:** Department of Human Oncology, University of Wisconsin Comprehensive Cancer Center, Madison 53792, USA.

## Abstract

**Images:**


British Journal of Cancer (1997) 75(1), 17–27
© 1997 Cancer Research Campaign

### Cloning and characterization of a 77-kDa oestrogen receptor isolated from a human breast cancer cell line

**JJ Pink[1], M Fritsch[1,*], MM Bilimoria,[2] VJ Assikis[2] and VC Jordan[1,2]**

[1]Department of Human Oncology, University of Wisconsin Comprehensive Cancer Center, Madison, Wisconsin 53792; [2]Robert H Lurie Cancer Center, Northwestern University Medical School, Chicago, Illinois 60611, USA

**Summary** We have cloned and characterized a 77-kDa oestrogen receptor (ER) from an oestrogen-independent subclone of the MCF-7 human breast cancer cell line. This receptor contains an in-frame, tandem duplication of exons 6 and 7, located in the steroid-binding domain of the ER. This mutation has abrogated ligand binding, but not DNA binding, in this mutant ER. We previously described the partial structure of a unique oestrogen receptor (ER) that is expressed in an oestrogen-independent MCF-7:2A subclone of the breast cancer cell line MCF-7 (Pink JJ, Wu SQ, Wolf DM, Bilimoria MM, Jordan VC 1996*a*, *Nucleic Acids Res* **24** 962–969). Sequence analyses determined the molecular weight of this 80-kDa ER to be 77 kDa, and hereafter this protein will be designated as $ER^{77}$. Examination of the entire coding sequence of the $ER^{77}$ mRNA indicates that it contains a tandem duplication of exons 6 and 7. Using a coupled transcription/translation system, a 77-kDa ER, which corresponds to the protein observed in the MCF-7:2A cells, was expressed. The $ER^{77}$ protein does not bind the ligands [$^3$H] oestradiol or [$^3$H]tamoxifen aziridine. In DNA binding gel shift assays, the in vitro synthesized $ER^{77}$ binds to a consensus vitellogenin $A_2$ oestrogen-response element. In transient transfection experiments, the mutant ER, alone or in combination with the wild-type ER, does not induce expression of an oestrogen-responsive luciferase reporter construct. In fact, expression of the $ER^{77}$ in the ER-positive T47D:A18 cell line inhibits $E_2$-induced luciferase expression. Overexpression of wild-type ER in T47D:A18 cells leads to elevated constitutive expression of the luciferase reporter, which was inhibited by co-transfection with $ER^{77}$. These data suggest that the $ER^{77}$ can interfere with normal ER activity and does not act as a constitutive activator of oestrogen-independent growth in MCF-7:2A cells. Consequently, the constitutive growth observed in MCF-7:2A cells is probably the result of other ER-mediated pathways.

**Keywords:** oestrogen receptor; breast cancer; exon duplication; MCF-7

The development of oestrogen-independent growth in previously oestrogen-dependent breast cancer is the single most significant problem in the clinical treatment of this disease (Paik et al, 1994; Paridaens, 1995). The oestrogen receptor (ER)-positive, oestrogen-responsive breast cancer cell line MCF-7 has been used as a model over the past 20 years (Brooks et al, 1973; Soule et al, 1973). More recently, these cells have been used to study the development of oestrogen-independent growth in breast cancer cells. The discovery in 1986 of the oestrogenic activity present in commercial preparations of the pH indicator, phenol red, and its subsequent removal from tissue culture media, has allowed the growth of cell lines in truly oestrogen-free media (Berthois et al, 1986). This finding led our laboratory, as well as others, to investigate the changes in the MCF-7 cell line that allowed these cells to adapt to growth in oestrogen-free media. Short-term studies showed that MCF-7 cells consistently expressed high levels of the ER following oestrogen removal, even as the growth of these cells became oestrogen independent. Interestingly, this basal growth in oestrogen-free media could be inhibited by a number of different anti-oestrogens (Katzenellenbogen et al, 1987; Welshons and Jordan, 1987). This phenotype was believed to be an intermediate step in the development of true anti-oestrogen resistance.

*Received 7 May 1996*
*Revised 10 July 1996*
*Accepted 12 July 1996*

*Correspondence to:* VC Jordan, Robert H Lurie Cancer Center, Northwestern University Medical School, 303 East Chicago Avenue, Olsen Pavilion 8258, Chicago, IL 60611, USA. *Present address: National Cancer Institute, Laboratory of Pathology, Building 10, Room 2N206, Bethesda, MD 20892, USA

In order to study the development of oestrogen-independent growth, we characterized a number of clones derived from MCF-7 cells, which were cultured in oestrogen-free media for more than 2 years. Each of these clones exhibited unique characteristics; however, they all continue to express high levels of active ER. One previously described clone, MCF-7:5C, became anti-oestrogen resistant, while continuing to express a functional wild-type ER (Jiang et al 1992*a*). Another oestrogen-independent clone, MCF-7:2A, isolated from the same parental MCF-7 cell line, continues to express wild-type ER. However, the growth of these cells is inhibited by anti-oestrogens of both the pure and partial agonist class (Pink et al, 1995).

In the present study, we describe MCF-7:2A cells, which express wild-type ER, in addition to a unique 77 (kDa) mutant ER ($ER^{77}$), which contains a tandem, in-frame duplication of exons 6 and 7 (Pink et al, 1996*a*). The expression of the 77-kDa ER in ten individual subclones of the MCF-7:2A cell line and its maintenance for over 100 passages suggests that it is critical to the oestrogen-independent growth of these cells (Pink et al, 1995). We now report the cloning and complete sequencing of the 77-kDa ER and a preliminary evaluation of the biology of the translated protein receptor. Functional characterization of this unique protein may offer insights into the mechanism responsible for the development of oestrogen-independent growth in breast cancer. These data may also shed light on at least one pathway that can lead to the development of oestrogen-independent and, possibly, anti-oestrogen-resistant growth in breast cancer.

### MATERIALS AND METHODS

### Cell culture

MCF-7 cells were obtained from Dean Edwards (at the San Antonio Breast Cancer Group, TX, USA) (originally obtained from the Michigan Cancer Foundation, Detroit, MI, USA). T47D (Keydar et al, 1979) and MDA-MB-231 (Caileau et al, 1974) cells were obtained from the American Type Culture Collection, Rockville, MD, USA All tissue culture components were obtained from Gibco Laboratories, Grand Island, NY, USA, unless otherwise stated. MCF-7:WS8, T47D:A18 and T47D:C4:2W cells were grown in RPMI-1640 containing 10% heat-inactivated fetal bovine serum (FBS; Bioproducts for Science, Indianapolis, IN, USA), 6 ng $ml^{-1}$ bovine insulin, 2 mM L-glutamine, 100 U $ml^{-1}$ penicillin, 100 μg $ml^{-1}$ streptomycin and 250 ng $ml^{-1}$ amphotericin B (fully oestrogenized medium). MCF-7:2A and MDA-MB-231: 10A cells were routinely grown in oestrogen-free medium, which substitutes phenol red-free RPMI and 3× dextran-coated charcoal-treated FBS. Cells were passed at 1:10–1:20 dilutions once per week using 0.1% trypsin.

MCF-7:WS8 is a clone of the MCF-7 cell line grown in oestrogen-containing medium. This cell line is dependent on oestrogen for maximum growth and ER-mediated gene expression (Pink et al, 1995). The clone MCF-7:2A was isolated from the same parental MCF-7 cell line following growth in oestrogen-free medium for more than 8 months (Jiang et al, 1992*a*). MCF-7:2A cells now grow maximally in oestrogen-free media; however, MCF-7:2A cells are inhibited by anti-oestrogens. The MCF-7:2A clone also expresses a mutant ER, which migrates at approximately 77 kDa. This mutant ER has been observed in ten individual subclones of this cell line and has been expressed for more than 100 passages (Pink et al, 1995). T47D:A18 is an ER- and progesterone receptor (PR)-positive, oestrogen-dependent clone derived from the T47D cell line. T47D: C4:2W is an ER- and PR-negative clone derived from T47D, following growth in oestrogen-free media for more than 1 year (Murphy et al, 1989, 1990; Pink et al, 1996*b*).

### Western blotting

Whole cell extracts were prepared by direct lysis of phosphate-buffered saline (PBS)-washed cells in 1 × sample buffer (10% glycerol, 150 mM Tris-HCI, pH 6.8, 0.5 mM EDTA, 0.125% sodium dodecyl sulphate (SDS), 1% β-mercaptoethanol and 5 μg $ml^{-1}$ bromphenol blue) followed by immersion in a boiling water bath for 5–10 min. Equal amounts of protein were run in a standard Western blot as described previously (Pink et al, 1995) with the following changes. The secondary antibody used was a horseradish peroxidase (HRP)-conjugated goat anti-rat antibody (HyClone Laboratories, Logan, UT, USA), and visualization was accomplished using the ECL visualization kit (Amersham Arlington Heights, IL, USA) according to the manufacturer's directions. The membrane was wrapped in plastic film and exposed to Kodak X-Omat film for 15s and developed.

### XL–PCR-mediated cloning of the wild-type ER and $ER^{77}$

Poly-$A^+$-enriched RNA was prepared from MCF-7:WS8 and MCF-7:2A cells grown in oestrogen-free medium by direct isolation (Badley et al, 1988). Poly $A^+$-enriched RNA, 5 μg per reaction, was reverse transcribed using Moloney murine leukaemia virus (MMLV) reverse transcriptase primed with oligo $(dT)_{12-18}$ (Gibco BRL, Gaithersburg, MD, USA). XL–PCR (Perkin-Elmer, Foster City, CA,USA) was then performed using 10% of this reaction and 100 ng of an upstream primer, which binds 10 bases 5′ of the translation start signal (U1, GCCACG-GACCATGACCATGA), and a downstream primer, which binds 3′ of the translation termination signal (D5, TGTGGGAGCCAGGGAGCTCT) (Oligos Etc., Wilsonville, OR, USA) as per the manufacturer's directions. Polymerase chain reaction (PCR) was run for a total of 31 cycles in a DNA thermal cycler (Perkin-Elmer-Cetus, Foster City, CA, USA), 1 min at 93°C, 12 min at 68°C for 16 cycles followed by 15 additional cycles as above, with a 15s per 68°C cycle extension and a final 20 min extension at 72°C. The PCR products were then blunt end ligated into the *Sma*I site of pUC18 using the Sure Clone Ligation kit (Pharmacia Biotech, Piscataway, NJ, USA). These clones were then digested with *Bam*HI to expose the 5′ end of the insert. The ends were filled using Klenow polymerase, and *Eco*RI linkers were ligated to the blunt ends. The full-length cDNA was liberated by digestion with *Eco*RI, gel purified and subcloned into the *Eco*RI site of pSG5 (Green et al, 1988). A sense and antisense clone of each ER cDNA was then selected for further characterization. The sequence of the entire cDNA inserts of both the wild-type ER and $ER^{77}$ were then determined using standard dideoxy chain termination methodology. The sequencing reaction was performed using Sequenase T7 DNA Polymerase (version 2.0, United States Biochemical, Cleveland, OH, USA) as per the manufacturer's instructions.

### Coupled in vitro transcription/translation of ER

The TNT Coupled Reticulocyte Lysate transcription/translation system (Promega Madison, WI, USA) was used to drive the synthesis of the ER proteins from the T7 promoter in pSG5 as per the manufacturer's directions. Radiolabelled protein was synthesized by including [$^{35}$S]methionine (Amersham) in the reaction mixture. These extracts were denatured by boiling in 1 × sample buffer and run on a 10% polyacrylamide SDS gel with a 4% stacking gel, fixed in 50% methanol–10% acetic acid and dried. The dried gel was exposed to radiographic film for 1 h and developed. The ER proteins were also synthesized in a reaction using only radioinert amino acids and run in a Western blot using the methods described above.

### Hydroxylapaptite binding assay

Oestradiol-binding assays were performed as described previously (Fritsch et al, 1993). Briefly, variable amounts of in vitro synthesized ERs were mixed with [$^{3}$H]17β-oestradiol ($E_2$) (40 nM final $E_2$ concentration) for 1 h at 4°C in 200 μl total volume. Hydroxylapatite (HAP) [0.25 ml of a 70% (v/v) slurry] was then added to this mixture along with 1 ml of 10 mM Tris, pH 7.5, and incubated at 4°C for 30 min, with three mixings. The HAP was pelleted and then washed four times with 2 ml of 10 mM Tris, pH 7.5. The bound [$^{3}$H]oestradiol was then eluted in 0.75 ml of 100% ethanol and counted in a Beckman LS 6000IC scintillation counter. To determine non-specific binding, the reaction was done in parallel with the addition of 8 μM diethylstilboestrol (DES), and specific binding was determined by subtracting non-specific counts from total counts.

© Cancer Research Campaign 1997

### [$^3$H]tamoxifen aziridine binding

This reaction was performed as described previously (Fritsch et al, 1992). Briefly, cells were grown in oestrogen-free media for 4 days and cytosols were prepared. The cytosols were then incubated with 120 nM [$^3$H]tamoxifen aziridine in a reaction either without (H) or with (HC) 6 μM radioinert DES for 90 min at 4°C. An equal volume of 2 × sample buffer was then added and the samples were boiled for 5 min. Amounts of cytosols containing equal [$^3$H]oestradiol binding, as measured in a HAP assay, were then separated by SDS-PAGE. The gel was fixed with 10% acetic acid and 50% methanol and then incubated with the fluor Enhance for 1 h at room temperature. Finally, the gel was washed with distilled water, dried and exposed to radiographic film at −70°C for 14 days.

### Gel shift assays

Gel shift assays were performed using the components provided in the BandShift kit (Pharmacia Biotech). All binding reactions contained 10 mM Tris-HCl (pH 7.5), 50 mM sodium chloride 3 mM dithiothreitol (DTT), 10% glycerol, 0.05% NP-40, 0.1 mM zinc chloride, 50 μg ml$^{-1}$ poly (dI-dC) and 1 ng of labelled oligonucleotide. Oligonucleotides were labelled with Klenow polymerase. Binding reactions were carried out overnight at 4°C or at room temperature for 1 h. For supershift experiments, 1 μg of the monoclonal antibody H222 (Greene et al, 1980) was added during the final 20 min of binding. Non-denaturing polyacrylamide gel electrophoresis (4%) was carried out in the cold using 1 × low ionic strength buffer (7 mM Tris HCl, pH 7.5, at 22°C, 3 mM sodium acetate and 1 mM EDTA) with constant buffer recirculation. Following electrophoresis, the gels were dried and exposed to radiographic film.

### Transient transfection assays

Cells were seeded into six-well plates (500 000 cells per well) in phenol red-free RPMI plus 10% 3 × charcoal-stripped FBS. The following day, medium was removed and replaced with fresh oestrogen-free medium. A solution containing 1 μg of the luciferase reporter construct, pVIT3-luc (Catherino and Jordan, 1995) and 0.5 μg of the β-galactosidase reporter, pCMVβ, plus the appropriate amounts of the ER expression constructs (MacGregor and Caskey, 1989) in 0.25 M calcium chloride was mixed dropwise with an equal volume of 2 × HBS (0.28 M sodium chloride, 0.05 M Hepes, 1.5 mM sodium phosphate, pH 7.05) by gently bubbling air through the solutions. Total DNA per group was equalized by including the pSG5 vector alone as carrier. This solution was then incubated at room temperature for 20 min to allow a DNA/calcium phosphate precipitate to form. This solution was slowly added to the cells and incubated at 37°C in a humidified incubator with 5% carbon dioxide for 6 h. At that time, the DNA solution was removed and medium, with or without compounds, was added to the wells and incubated at 37°C in a humidified 5% carbon dioxide incubator for an additional 18–48 h. The medium was removed and the cells were washed once with ice-cold PBS. The cells were then scraped in extraction buffer (0.1 M potassium hydrogen phosphate, pH 7.5, 1% Triton X-100, 100 μg ml$^{-1}$ bovine serum albumin (BSA), 2.5 mM phenylmethylsulphonyl fluoride (PMSF) and 1 mM DTT) and pipetted vigorously to ensure complete cell lysis. Cell debris was pelleted by spinning in a microfuge for 1 min and the lysate was stored on ice until luciferase activity was assayed. Luciferase activity was assayed by mixing 50 μl of each lysate with 350 μl of reaction buffer (160 mM magnesium chloride, 75 mM glycylglycine, pH 7.8, 0.5 mg ml$^{-1}$ BSA, 19 mg ml$^{-1}$ ATP and 15 mM Tris-HCl, pH 7.5). To begin each assay, 100 μl of substrate (0.4 mg ml$^{-1}$ luciferin, potassium salt in 10 mM sodium bicarbonate, pH 6.0) was automatically injected into the lysate mixture. Each point was monitored for 10 s by a Monolight 2010B luminometer (Analytical Luminescence Laboratory, San Diego, CA, USA) and relative luciferase units (RLUs) were then reported. All points were corrected for transfection efficiency by dividing RLU by β-galactosidase activity.

β-Galactosidase activity was measured using a β-methylumbelliferone (MUG) assay (Luyten et al, 1985). Briefly, an aliquot of the cell extract was mixed with 1.3 ml of reaction buffer containing 0.1 M sodium phosphate, 10 mM potassium chloride, 1 mM magnesium sulphate, pH 7.0, and 2.2 ($10^{-5}$) g ml$^{-1}$ MUG (Molecular Probes, Eugene, OR, USA). The sample was incubated at room temperature for 1 h and 750 μl of stop buffer (15 mM EDTA, 0.3 M glycine, pH 11.2) was added. The samples were then read in an LS-5 fluorescence spectrophotometer (Perkin Elmer, Foster City, CA, USA) with excitation at 350 nm and absorption at 450 nm. All samples were correlated to a standard curve using purified β-galactosidase (Boehringer Mannheim Biochemicals, Indianapolis, IN, USA)

### RESULTS

### Cloning of the wild-type ER and ER$^{77}$ full-length cDNAs

Previously, we showed that the mutant ER isolated from the MCF-7:2A cells contains an in-frame duplication of exons 6 and 7. This was determined by partial PCR-mediated subcloning of a segment of the ER cDNA, which comprised approximately one-half of the coding sequence of the mutant ER (Pink et al, 1995). Calculation of the molecular weight of this protein based upon the amino sequence resulted in an estimate of 77 kDa. This is in good agreement with our initial estimate of 80 kDa, which was based solely on migration in SDS-PAGE. We have designated this mutant ER as ER$^{77}$ for all future reference. A reverse transcriptase–polymerase chain reaction (RT–PCR)-mediated approach was subsequently used to clone the full-length cDNA for the mutant ER$^{77}$, as well as the wild-type 66-kDa ER present in the MCF-7:2A cell line. PCR amplification of a cDNA library from MCF-7:2A cells gave rise to two major amplified products of approximately 1800 bp and approximately 2100 bp. These blunt-ended products were cloned into the *SmaI* site of pUC18. Clones were then screened by PCR to isolate plasmids containing the correct inserts. Clones containing the wild-type ER (1800 bp) and ER$^{77}$ (2100 bp) inserts were then prepared and sequenced using standard dideoxynucleotide methodology, as described in Materials and methods. These inserts were subcloned into the eukaryotic expression vector pSG5 (Green et al, 1988) for further examination. The coding sequence of ER$^{77}$ is shown in Figure 1, with the duplicated exons highlighted. Sequence analysis of the wild-type ER (sense orientation pSG5:58–1; antisense orientation pSG5:58–3) showed no mutations in the coding sequence when compared with the normal ER (HEGO) sequence, as described by Tora et al (1989). The ER$^{77}$ cDNA clones (sense orientation pSG5:91A; antisense orientation pSG5:91C) were also sequenced and shown to contain a duplication of exons 6 and 7 as described previously. No other alterations were observed in the entire cDNA coding sequence.

© Cancer Research Campaign 1997

**Figure 1** Coding sequence of the mutant $ER^{77}$. Numbers refer to the amino acids sequence. Exon boundaries are as previously reported (Ponglikitmongkol et al, 1988). The shaded region corresponds to the duplicated exons 6 and 7

*© Cancer Research Campaign 1997*

**Figure 2** Translation of ER cDNAs in a reticulocyte lysate system. Equal volumes of TNT coupled lysates programmed with pSG5-derived expression vectors were denatured, run on an SDS polyacrylamide gel and probed in a Western blot using the anti-ER antibody, H222. HEGO is the wild-type ER cDNA originally described by Tora et al (1989). The wild-type ER and $ER^{77}$ are ER cDNA clones isolated from MCF-7:2A cells, and these inserts in the antisense orientation are also shown

### Coupled in vitro transcription/translation of the ER cDNAs

The pSG5 vector contains a T7 promoter upstream of the multiple cloning site into which the ER cDNAs were cloned (Green et al, 1988). We tested the ability of wild-type ER and $ER^{77}$ constructs to code for protein that could be used to assess the biochemical properties of these ERs. A reaction that allowed for the incorporation of [$^{35}$S]methionine was performed, and a quantity of that reaction was then run on a standard SDS-polyacrylamide gel. HEGO and wild-type ER translation gave rise to a single major band of approximately 66 kDa. The wild-type ER and $ER^{77}$ antisense clones did not yield any $^{35}$S-labelled proteins, as expected (data not shown). Translation of the $ER^{77}$ clone demonstrated a major $^{35}$S-labelled protein at 77 kDa. This reaction was repeated with radioinert methionine, and the proteins were probed in a Western blot using the monoclonal anti-ER antibody, H222. As observed in Figure 2, the major protein species visualized by SDS-PAGE were recognized by this specific antibody. The minor bands seen in the Western blot are probably the result of either proteolytic cleavage, an internal codon that causes premature termination of translation or, alternatively, a cryptic initiation site upstream of the canonical start site that gives rise to truncated proteins that still contain the epitope recognized by the H222 antibody. These minor bands constitute less than 2% of total ER synthesis and should not significantly affect the functional studies described below. These data confirm that the clone, pSG5:58–1, codes for a wild-type ER, and pSG5:91A codes for the $ER^{77}$, as first observed in the MCF-7:2A cells.

**Figure 3** Ligand binding of the wild-type and mutant ERs. (**A**) $E_2$ binding of in vitro synthesized ERs was measured in a HAP binding assay. See Materials and methods for details. The average binding of four volumes of lysate is shown. Groups are as described in Figure 2. (B) Binding of ER isolated form MCF-7:WS8 and MCF-7:2A cells to [$^3$H]tamoxifen aziridine. Cytosols containing equal amounts of ER were mixed with [$^3$H]tamoxifen aziridine for 90 min at 4°C alone (H) or in the presence of a 200-fold molar excess of DES (HC). The samples were then denatured and run in a standard SDS polyacrylamide gel, dried and exposed to radiographic film

### Ligand binding of the $ER^{77}$

Following reticulocyte synthesis, wild-type and mutant ERs were incubated in reactions containing 40 nM[$^3$H]$E_2$ at 4°C for 1 h. Specific binding of [$^3$H]$E_2$ was then measured by HAP binding. Non-specific binding was measured in parallel groups containing a 200-fold molar excess of unlabelled DES. The results of this experiment are shown in Figure 3A. For each group 1, 5, 10 and 25 μl of extract was incubated with the [$^3$H]$E_2$. Total specific binding in each reaction was measured and specific binding per μl extract was calculated from each reaction. Both HEGO and the wild-type ER isolated from MCF-7:2A cells bind [$^3$H]$E_2$. Neither

© Cancer Research Campaign 1997 

**Figure 4** Specific DNA binding of ERs isolated from the MCF-7:WS8 and MCF-7:2A cell lines. Nuclear extracts were isolated from oestrogen-deprived MCF-7:WS8 and MCF-7:2A cells. The indicated amount of each extract was then mixed with a $^{32}$P-labelled 20-bp oligonucleotide containing the vitellogenin $A_2$ ERE for 30 min at 20°C. The anti-ER antibody, H222, was then added to the appropriate groups, and all reactions were incubated for an additional 30 min at 20°C. The reactions were then run on a 4.5% non-denaturing polyacrylamide gel and visualized by exposure to radiographic film

**Figure 5** Specific DNA binding of ERs synthesized in vitro. Reticulocyte lysate-synthesized ERs were mixed with a $^{32}$P-labelled 60-bp oligonucleotide containing three copies of the vitellogenin $A_2$ ERE. Following incubation at 4°C overnight, H222 antibody was added to the appropriate groups and incubated for 30 min at 20°C. These reactions were then analysed as described for Figure 4. The addition of 1 μg of H222 is noted by a (+). The addition of a 100 × molar excess of cold ERE is noted in the last two lanes of the antisense programmed extracts demonstrated any specific $E_2$ binding. The $ER^{77}$-containing extract failed to demonstrate any specific ligand binding in this assay.

The ability of the 66-kDa and 77-kDa ER to bind ligand was also measured in cytosols from MCF-7:2A and MCF-7:WS8 cells by means of covalent binding to [$^3$H]tamoxifen aziridine (Wei et al, 1985). Cytosols from both MCF-7:WS8 and MCF-7:2A cells, grown for 4 days in oestrogen-free medium, were incubated with [$^3$H]tamoxifen aziridine in the presence (HC) and absence (H) of a 200-fold molar excess of DES. As seen in Figure 3B, both cell lines show specific binding of a 66-kDa species that was competed by DES. However, MCF-7:2A cells did not show binding to any species that corresponded to $ER^{77}$. Together, these data demonstrate that the exon duplication in the ligand-binding domain of the $ER^{77}$ protein has abrogated ligand-binding ability.

### DNA binding of the $ER^{77}$

Gel shift analyses of nuclear extracts from the MCF-7:2A cells did not demonstrate any obvious higher molecular weight species that could be the result of specific binding of the $ER^{77}$ to a 20-bp oligonucleotide, which contained a consensus vitellogenin $A_2$ oestrogen-response element (ERE) (Figure 4). The MCF-7:2A cytosol demonstrated higher binding per μl of extract as would be expected from its approximately two fold higher wild-type ER expression. In order to measure the DNA binding of the $ER^{77}$ in the absence of the wild-type ER, we repeated this assay using the in vitro synthesized $ER^{77}$. For this assay, we used a 60-bp oligonucleotide that contained three copies of the vitellogenin $A_2$ ERE separated by *Hind*III sites. Use of this oligo gave rise to much stronger gel-shifted bands of similar mobility to those observed with the 20-bp oligo containing a single ERE (data not shown). Total $^{35}$S protein labelling showed that the reticulocyte extracts

© Cancer Research Campaign 1997

**Figure 6** Transcriptional activity of transiently transfected ERs. pVIT3-Luc reporter (1 μg) was transfected into T47D:A18 (**A** and **B**) or T47D:C4:2W (**C** and **D**) cells using a standard calcium phosphate technique, along with 0.5 μg of the pCMVβ β-galactosidase plasmid, which served as a transfection control. Included in these reactions was the noted quantity of the vectors coding for the wild-type ER or $ER^{77}$. Six hours after the transection, fresh oestrogen-free medium (**A** and **C**) or medium containing 1 nM $E_2$ (**B** and **D**) was added. The cells were analysed for luciferase activity and β-galactosidase 42 later. All results were corrected for β-galactosidase activity and presented as fold induction relative to cells with no exogenous ER expression vectors

© Cancer Research Campaign 1997 

expressed approximately 25% less $ER^{77}$ than HEGO protein per µl of extract. Western analyses demonstrated approximately 50% less $ER^{77}$ than wild-type ER when measured by H222 binding (see Figure 2). DNA binding of the in vitro synthesized ERs are shown in Figure 5. The wild-type ER demonstrated specific ERE binding, which was supershifted by the ER-specific antibody, H222. The $ER^{77}$ extract gave rise to a shifted complex, which ran as a smear from a site similar to that of the wild-type ER complex to a site considerably higher in the gel. The H222 antibody specifically bound to the $ER^{77}$ complex and gave rise to a supershifted complex that was clearly larger than that observed with wild-type ER. The specificity of the DNA binding was shown by the ability of an excess of cold ERE to inhibit the binding to the radiolabelled ERE completely. In mixing experiments designed to assess the interaction of the wild-type ER and $ER^{77}$, no evidence of interaction or heterodimerization of the two receptors has been observed (data not shown).

### Transient transfection studies

We next performed transient transfection studies in order to determine the ability of wild-type ER and $ER^{77}$ to induce transcription from an oestrogen-responsive promoter. For these studies, we used a luciferase reporter construct, pVIT3-Luc, which contained the same 60-bp oligonucleotide insert used in the DNA-binding studies upstream of a minimal herpes simplex thymidine kinase promoter. In previous studies, we demonstrated that this construct was exquisitely sensitive to ER-mediated transcription (Catherino and Jordan, 1995). The pSG5 (Green et al, 1988)-derived ER expression vectors contain the SV40 early promoter, a rabbit β-globin intron II and an SV40 poly-A signal, which afforded constitutive expression of these proteins. The cell lines that we chose for these transfection studies are an ER-positive (T47D:A18) and an ER-negative (T47D:C4:2W) clone of the human breast cancer cell line, T47D. Comparable ER-positive and -negative clones are not available for the MCF-7 cell line. The T47D:A18 clone exhibits a dramatic growth response to oestrogens, which can be inhibited by antio-estrogens. The T47D:C4:2W clone was derived from the T47D cell line by long-term (>2 years) growth in oestrogen-free media, and two rounds of limiting dilution cloning. This clone is ER negative and completely devoid of any measurable response to either oestrogens or anti-oestrogens (Pink et al, 1996*b*).

In the absence of exogenous ER, T47D:A18 cells display an approximately 100-fold induction of luciferase activity in response to oestradiol, as seen in lane 5 of Figure 6B. Addition of the wild-type ER expression vector caused an approximately 50% increase in this activity. However, co-transfection of $ER^{77}$ with the wild-type ER clearly caused a decrease in luciferase activity. Additionally, transfection with the $ER^{77}$ alone causes an even greater inhibition of the endogenous oestradiol-induced luciferase activity in T47D:A18 cells (see lanes 7 and 8 of Figure 6B). In a parallel experiment designed to assess the oestrogen-independent function of the ER in T47D:A18 cells, transfection of wild-type ER caused an approximately 15-fold induction of luciferase compared with T47D:A18 cells alone, (see lane 2 of Figure 6B). This oestrogen-independent activity was not observed in the $ER^{77}$-transfected group. In fact, as seen in lane 3 of Figure 6B, the $ER^{77}$ inhibits the oestrogen-independent activity of the wild-type ER.

T47D:C4:2W cells do not show significant oestrogen-independent luciferase activity in any group, as shown in Figure 6C. However, transfection of wild-type ER in these cells resulted in an approximately 15-fold induction of luciferase activity in the presence of $E_2$. In contrast to the T47D:A18 cells, $ER^{77}$ in oestrogen-free medium had no activity in the T47D:C4:2W cells (see Figure 6). However, $ER^{77}$ inhibited the oestradiol-induced wild-type ER activity when these two genes were co-transfected (compare lanes 6 and 7 in Figure 6D).

### DISCUSSION

We used the MCF-7 cell line as a model system in order to examine the adaptation of oestrogen-dependent breast cancer cells to growth in oestrogen-free media. This cell line maintained oestrogen-responsive growth when continuously cultured in oestrogen-containing media. However, when cultured in oestrogen-free media, MCF-7 cells underwent a slowing of growth and a subsequent crisis period, during which many of the cells died (Katzenellenbogen et al, 1987; Welshons and Jordan, 1987). Following this crisis, clones appeared that grew well in oestrogen-free media. These adapted cells all appear to share two characteristics. First, they continue to express the ER. Second, their oestrogen-independent growth can be inhibited by anti-oestrogens. From these oestrogen-independent MCF-7 cells, we isolated the MCF-7:2A clone following two rounds of limiting dilution cloning. While maintaining ER expression and sensitivity to the growth-inhibitory effects of anti-oestrogens, MCF-7:2A cells express a novel 77-kDa ER, in addition to the wild-type 66-kDa ER (Pink et al, 1995, 1996*a*). Prior investigation of the function of the mutant $ER^{77}$ in MCF-7:2A cells was problematic owing to the masking of the $ER^{77}$ function by the wild-type ER. In MCF-7:2A cells, the wild-type ER is expressed at three to ten fold greater levels than that of the mutant receptor. In order to circumvent this problem and to study the independent function of this mutant ER, we cloned the full-length cDNA, which coded for the $ER^{77}$. Previously, we had cloned and sequenced a PCR-generated fragment of $ER^{77}$, which contained a duplication of exons 6 and 7 (Pink et al, 1996*a*). However, this fragment could not be used to prepare a full-length cDNA for functional studies of the $ER^{77}$, and there was no evidence from our previous studies that point mutations were not present elsewhere in the molecule. In the present study, we demonstrate that the only alteration in the coding sequence of the $ER^{77}$ cDNA was the tandem duplication of exons 6 and 7. We also used XL–PCR to clone the cDNA for the wild-type ER from the MCF-7:2A cells to determine whether this receptor contained any mutations in its coding sequence. Previously, our only evidence that the wild-type ER from the MCF-7:2A cells was normal was based on its apparent size (Pink et al, 1995, 1996*a*). Here, we showed that the 66-kDa ER does not contain any mutations in its coding sequence. This suggests that the abnormal ER pathway present in the MCF-7:2A cells is not the result of previously undetected mutations in the 66-kDa ER. The presence of the mutant $ER^{77}$ in all ten subclones studied so far suggests that this protein is associated with the development of the oestrogen-independent phenotype observed in the MCF-7:2A cells. However, this does not indicate that the presence of the $ER^{77}$ is the only defect in the ER-mediated signalling pathway; other alterations may subsequently prove to be critical factors in the development of oestrogen-independent growth in these unique cells.

Mutant and wild-type cDNA clones were further characterized by synthesizing the ERs in an in vitro coupled reticulocyte transcription/translation system. As we showed in Figure 2, the pSG5:58–1 clone expressed a 66-kDa ER, which is indistinguishable from the

© Cancer Research Campaign 1997

ER coded by the HEGO clone isolated by Green et al (1988). The pSG5:91A clone, which contains the cDNA expressing $ER^{77}$ demonstrated production of a single protein of expected size that was recognized by two monoclonal antibodies to the ER (Figure 2 and data not shown). Addition of the antisense cDNAs in the reticulocyte lysates did not give rise to any proteins, as measured by either [$^{35}$S]methionine incorporation or Western blotting.

The ER has three primary functions that can be measured experimentally (Green and Chambon, 1991): (1) ligand binding; (2) specific DNA binding; and (3) transcriptional activation of responsive genes. We measured these three functions using both the wild-type ER and $ER^{77}$ isolated from the MCF-7:2A cells. The ligand-binding ability of these ERs was measured in two assays. Firstly, we measured the ability of the ERs synthesized in vitro to bind [$^{3}$H]$E_2$ in a HAP assay. As shown in Figure 3A, extracts containing the wild-type ER specifically bound to this ligand with a capacity equal to that of the HEGO-generated ER. In contrast, the $ER^{77}$ extracts did not measurably bind this ligand, showing no more binding than the antisense extracts. These studies were confirmed in an experiment that measured the ability of these receptors to bind the anti-oestrogenic ligand [$^{3}$H]tamoxifen aziridine covalently (Wei et al, 1985; Fritsch et al, 1993). As seen in Figure 3B, MCF-7:WS8 cytosol showed specific binding of a protein that migrated at approximately 66 kDa. The MCF-7:2A cytosol also contained a DNA-binding protein that migrated at approximately 66 kDa, as would be expected from the wild-type receptor identified in these cells. However, the MCF-7:2A cytosol did not demonstrate any specific binding corresponding to the $ER^{77}$. These results could be predicted based upon previous data, which have shown that point mutations in the steroid-binding domain of the ER can cause alterations in ligand-binding ability (Jiang et al, 1992*b*: Ince et al, 1993; Sluyser, 1995). We hypothesize that the duplication of exons 6 and 7, which causes the addition of over 100 amino acids to the ligand-binding domain of the ER, would have dramatic effects on the ligand-binding function of the $ER^{77}$ protein. We show here that this effect is to abolish any specific ligand binding in $ER^{77}$.

The DNA binding of the ER from these cells was measured in standard gel shift assays. In studies using nuclear extracts from MCF-7:2A cells, the binding of the wild-type 66-kDa ER is easily measured. However, even in lysates that have been depleted of the wild-type ER by ICI 182 780 exposure, the $ER^{77}$ does not appear to bind the vitellogenin $A_2$ ERE with a high affinity (Pink and Jordan 1996 and data not shown). We therefore used the receptor synthesized in vitro to demonstrate DNA binding by the $ER^{77}$ in isolation. Wild-type ER bound this ERE well in the absence of ligand; however, the addition of any ligand demonstrably increases DNA binding regardless of whether the ligand is an oestrogen or anti-oestrogen (see Figure 4). For the gel shift experiment observed in Figure 5, we used unoccupied receptor to equalize the DNA binding of the $ER^{77}$ and the wild-type ER. We reasoned that the lack of ligand-binding capability in the $ER^{77}$ would serve to keep this receptor in the 'unoccupied' state regardless of the presence of ligand. The presence of ligand in the wild-type ER reactions would, therefore, be expected to increase binding and overwhelm the binding of the $ER^{77}$. Lane 1 of Figure 5 shows that the unoccupied wild-type ER specifically binds this ERE, and lane 2 shows that the antibody H222 can supershift this complex to completion.

Interestingly, the $ER^{77}$ alone resulted in a complex, which appeared as a poorly defined smear that ran with mobility similar to the wild-type ER complex. The addition of the antibody, H222, appears to have two important effects on the $ER^{77}$ complex. Firstly, the supershift demonstrates that this antibody can still recognize the $ER^{77}$/DNA complex. Secondly, H222 binding appears to stabilize the DNA binding of $ER^{77}$, as demonstrated by a significant increase in signal intensity. The nature of the antibody stabilization of the $ER^{77}$ DNA binding is unclear. Furthermore, the cause of the diffuse pattern of the $ER^{77}$ binding was equivocal and may be the result of any number of factors, such as modification of the $ER^{77}$ protein following translation in the reticulocyte extract or possibly some form of oligomerization with other proteins present in the extract. The duplicated segment present in $ER^{77}$ may serve as a site for specific modification not present in the wild-type ER. The expression of the $ER^{77}$ protein in bacterial or baculovirus systems would address this possibility. The interaction of the $ER^{77}$ and the wild-type ER was also assessed in experiments in which various ratios of lysates containing the wild-type ER and/or the $ER^{77}$ were mixed and analysed in gel shift assays. In these experiments, there was no indication of bands of intermediate mobility, which would be the result of heterodimerization of the two receptors, as has been shown previously by Kumar and Chambon (1988) and data not shown.

The final ER function that was measured was the ability to transactivate transcription of a gene under the control of an ERE. For this analysis, we employed two cell lines derived from the well-studied human breast cancer cell line, T47D. T47D:A18 is a subclone of T47D, which is ER positive and oestrogen dependent. T47D:C4:2W is a clone, which was derived following long-term growth in oestrogen-free media and now grows maximally in oestrogen-free media, and is ER negative (Pink et al, 1996*b*). The use of these cell lines allowed us to measure the effect of the $ER^{77}$ on both the endogenously and exogenously expressed ER. We do not have an ER-negative clone derived from MCF-7 breast cancer cells, so comparable experiments are not possible. As a target of the transactivation, we chose a luciferase reporter system that was under the control of three copies of the *Xenopus* vitellogenin $A_2$ ERE. Data in Figure 6 showed that transfection of the wild-type ER lead to oestrogen-dependent induction of luciferase in the ER-negative T47D:C4:2W cells. T47D:A18 cells exhibit this same oestrogen-inducible response in the absence of transfected ER owing to endogenously expressed ER. In this assay, $ER^{77}$ does not cause either constitutive or oestrogen-induced luciferase activation. In fact, $ER^{77}$ inhibited the activity of both the exogenous and endogenous ER (compare lanes 4 and 8 in Figure 6A–D). In co-transfection groups, we chose a 1:5 ratio of $ER^{77}$ to wild-type ER in an attempt to mimic the ratio observed in MCF-7:2A cells. These groups demonstrated that the $ER^{77}$ can inhibit the transactivation capability of the wild-type ER.

An interesting result was obtained when oestrogen-free T47D:A18 cells were transfected with wild-type ER alone. These cells showed an approximately 15-fold increase in luciferase activity. This response was also observed in transfections with the MCF-7:WS8 cells (data not shown). However, when $ER^{77}$ was used, there was no increase in activity and the addition of $ER^{77}$ to the wild-type ER transfection caused an approximately 50% decrease in the constitutive activity. The wild-type ER-mediated $E_2$-independent activity is not observed in T47D:C4:2W cells. This suggests that in T47D:A18 cells, the constitutive expression of exogenous ER can lead to the induction of transcription in oestrogen-free media. This activity can be inhibited by co-transfection with $ER^{77}$ in T47D:A18 cells. $ER^{77}$ also inhibits endogenous $E_2$-stimulated activity in T47D:A18 cells, while co-transfection with wild-type ER caused a 50% increase in luciferase activity.

© Cancer Research Campaign 1997 

The induction of luciferase activity in T47D:C4:2W cells observed in Figures 6C and D is quite different. Transfection of the wild-type ER, mutant ER or a combination of these ERs showed no induction of luciferase activity in the oestrogen-free groups. In the $E_2$-treated groups, transfection of wild-type ER induced a >15-fold induction in luciferase activity. In T47D:C4:2W cells, which express no endogenous ER, the $ER^{77}$ did not induce any luciferase activity in response to $E_2$. As observed in the T47D:A18 groups, the addition of $ER^{77}$ to the wild-type ER caused an inhibition of luciferase activity below that seen with wild-type ER alone. These results show that $ER^{77}$ cannot function as a transcriptional activator of ERE-containing promoters and suggests that this mutant ER is an inhibitor of normal ER function. Further investigation will be necessary in order to elucidate fully the interaction of the $ER^{77}$ with the wild-type ER, as well as with other transcriptionally active nuclear proteins.

Initially, we believed that the $ER^{77}$ protein was responsible for the oestrogen-independent growth and transcriptional activity observed in the MCF-7:2A cells. The data presented here do not support this position. In contrast, we found that increasing the amount of ER in the T47D:A18 cells, through the use of an exogenous expression system, caused an increase in the oestrogen-independent activation of the luciferase reporter gene. This suggests that the mechanism for the oestrogen-independent growth in the MCF-7:2A cells may be the result of the increased expression of wild-type 66-kDa ER. Elevated expression of wild-type ER may serve to activate genes, which are responsible for the growth of the MCF-7:2A cell line in the absence of oestrogens. This may be due simply to a mass action effect or, possibly, a non-ligand-mediated activation pathway (Cho and Katzenellenbogen, 1993) that allows the ER to drive growth but not endogenous ER-responsive genes, such as the PR. Alternatively, MCF-7:2A cells may have adapted to growth in oestrogen-free media through the activation of a pathway that caused activation of the $ER^{77}$ through a mechanism, which is not observed in transient transfection studies. We propose that the $ER^{77}$ may serve as a brake to prevent that overstimulation of the growth pathways in these cells. This is supported by the observation that oestrogen does not increase the growth of the MCF-7:2A cells and, in fact, causes a slight decrease in growth in a 6-day assay (Pink et al, 1995). It seems likely that the MCF-7:2A cells have developed a delicate balance of oestrogen-independent, ER-mediated activity that allows them to proliferate in the absence of exogenous oestrogens. Any perturbation of this balance by either oestrogen or anti-oestrogens causes a slowing of growth. Further investigations, including stable expression of the $ER^{77}$ protein in previously ER-negative cells, will be necessary to elucidate the mechanism of action of this mutant ER and its potential involvement in the development of oestrogen-independent growth.

### ACKNOWLEDGMENTS

This work was supported by NIH grant CA32713 to VCJ, and JJP was supported in part by NIH training grant 5T32-CA09471. MMB and VJA were supported by generous training fellowships from the Lynn Sage Breast Cancer Foundation. We thank Professor Pierre Chambon for the ER cDNA expression vector, HEGO, and Abbot Laboratories for the monoclonal ER antibody, H222. We thank Dr David Boothman for helpful discussions and critical reading of the manuscript. We thank Jay Pink, Pharmacia Biotech, for helpful discussions regarding PCR, cloning and sequencing. We also thank Matt Bong, Jim Holsen, Kyle Hansen and Michelle Mucks for excellent technical assistance.

### REFERENCES

Badley JE, Bishop GA, St John T and Frelinger JA (1988) A simple, rapid method for the purification of poly A+ RNA. *Biotechniques* **6:** 114–116

Berthois Y, Katzenellenbogen JA and Katzenellenbogen BS (1986) Phenol red in tissue culture media is a weak estrogen: implications concerning the study of estrogen-responsive cells in culture. *Proc Natl Acad Sci USA* **83:** 2496–2500

Brooks SC, Locke ER and Soule HD (1973) Estrogen receptor in a human cell line (MCF-7) from breast carcinoma. *J Biol Chem* **248:** 6251–6253

Caileau R, Young R, Olive M and Reeves WJJ (1974) Breast tumor cell lines from pleural effusions. *J Natl Cancer Inst* **53:** 661–674

Catherino WH and Jordan VC (1995) Increasing the number of tandem estrogen response elements increases the estrogenic activity of a tamoxifen analogue. *Cancer Lett* **92:** 39–47

Cho H and Katzenellenbogen BS (1993) Synergistic activation of estrogen receptor-mediated transcription by estradiol and protein kinase activators. *Mol Endocrinol* **7:** 441–452

Fritsch M, Anderson I and Gorski J (1993) Structural characterization of the trypsinized estrogen receptor. *Biochemistry* **32:** 14000–14008

Fritsch M, Leary CM, Furlow JD, Ahrens H, Schuh TJ, Mueller GC and Gorski J (1992) A ligand-induced conformational change in the estrogen receptor is localized in the steroid binding domain. *Biochemistry* **31:** 5303–5311

Green S and Chambon P (1991) The oestrogen receptor: from perception to mechanism. In *Nuclear Hormone Receptors: Molecular Mechanisms, Cellular Functions, Clinical Abnormalities.* Parker MG (ed.) pp. 15–37. Academic Press: London.

Green S, Issemann I and Sheer E (1988) A versatile in vivo and in vitro eukaryotic expression vector for protein engineering. *Nucleic Acids Res* **16:** 369

Greene GL, Fitch FW and Jensen EV (1980) Monoclonal antibodies to estrophilin: probes for the study of estrogen receptors. *Proc Natl Acad Sci USA* **77:** 157–161

Ince BA, Zhuang Y, Wrenn CK, Shapiro DJ and Katzenellenbogen BS (1993) Powerful dominant negative mutants of the human estrogen receptor. *J Biol Chem* **268:** 14026–14032

Jiang SY, Wolf DM, Yingling JM, Chang C and Jordan VC (1992*a*) An estrogen receptor positive MCF-7 clone that is resistant to antiestrogens and estradiol. *Mol Cell Endocrinol* **90:** 77–86

Jiang SY, Langan FS, Stella AL, McCague R and Jordan VC (1992*b*) Point mutation of estrogen receptor (ER) in the ligand-binding domain changes the pharmacology of antiestrogens in ER-negative breast cancer cells stably expressing complementary DNAs for ER. *Mol Endocrinol* **6:** 2167–2174

Katzenellenbogen BS, Kendra KL, Norman MJ and Berthois Y (1987) Proliferation, hormonal responsiveness, and estrogen receptor content of MCF-7 human breast cancer cells grown in the short-term and long-term absence of estrogens. *Cancer Res* **47:** 4355–4360

Keydar I, Chen L, Karby S, Weiss FR, Delarea J, Radu M, Chaitcik S and Brenner HJ (1979) Establishment and characterization of a cell line of human breast cancer origin. *Eur J Cancer* **15:** 659–670

Kumar V and Chambon P (1988) The estrogen receptor binds tightly to its responsive element as a ligand-induced homodimer. *Cell* **55:** 145–156

Luyten GP, Hoogeveen AT and Galjaard H (1985) A fluorescence staining method for the demonstration and measurement of lysosomal enzyme activities in single cells. *J Histochem Cytochem* **33:** 965–968

MacGregor GR and Caskey CT (1989) Construction of plasmids that express *E.coli* beta-galactosidase in mammalian cells. *Nucleic Acids Res* **17:** 2365

Murphy CS, Meisner LF, Wu SQ and Jordan VC (1989) Short-and long-term estrogen deprivation of T47D human breast cancer cells in culture. *Eur J Cancer Clin Oncol* **25:** 1777–1788

Murphy CS, Pink JJ and Jordan VC (1990) Characterization of a receptor-negative, hormone-nonresponsive clone derived from a T47D human breast cancer cell line kept under estrogen-free conditions. *Cancer Res* **50:** 7285–7292

Paik S, Hartmann DP, Dickson RB and Lippman ME (1994) Antiestrogen resistance in ER positive breast cancer cells. *Breast Cancer Res Treat* **31:** 301–307

Paridaens, R (1995) Hormonal resistance in breast cancer. In *Drug and Hormonal Resistance in Breast Cancer: Cellular and Molecular Mechanisms.* Dickson RB and Lippman ME (eds) pp. 21–37. Ellis Horwood: Hemel Hempstead, UK.

Pink JJ, Jiang SY, Fritsch M and Jordan VC (1995) An estrogen independent MCF-7 breast cancer cell line which contains a novel 80 kilodalton estrogen receptor related protein. *Cancer Res* **55:** 2583–2590

© Cancer Research Campaign 1997

Pink JJ and Jordan VC (1996) Models of estrogen receptor regulation by estrogens and antiestrogens in breast cancer cell lines. *Cancer Res* **56:** 2321–2330

Pink JJ, Wu SQ, Wolf DM, Bilimoria MM and Jordan VC (1996*a*) A novel 80 kilodalton estrogen receptor containing a duplication of exons 6 and 7. *Nucleic Acids Res* **24:** 962–969

Pink JJ, Billimoria MM, Assikis VJ and Jordan VC (1996*b*) Irreversible loss of the estrogen receptor in T47D breast cancer cells following prolonged estrogen deprivation. *Br J Cancer* **74:** 1227–1236

Ponglikitmongkol M, Green S and Chambon P (1988) Genomic organization of the human oestrogen receptor gene. *EMBO J* **7:** 3385–3388

Sluyser M (1995) Mutations in the estrogen receptor gene. *Hum Mutat* **6:** 97–103

Soule HD, Vasquez J, Long A, Albert S and Brennan M (1973) A human cell line from a pleural effusion derived from a breast carcinoma. *J Natl Cancer Inst* **51:** 1409–1416

Tora L, Mullick A, Metzger D, Ponglikitmongkol M, Park I and Chambon P (1989) The cloned human oestrogen receptor contains a mutation which alters its hormone binding properties. *EMBO J* **8:** 1981–1986

Wei LL, Mangel WF and Katzenellenbogen BS (1985) Biological activities of tamoxifen aziridine, an antiestrogen-based affinity label for the estrogen receptor, in vivo and in vitro. *J Steroid Biochem* **23:** 875–881

Welshons WV and Jordan VC (1987) Adaptation of estrogen-dependent MCF-7 cells to low estrogen (phenol red-free) culture. *Eur J Cancer Clin Oncol* **23:** 1935–1939

© Cancer Research Campaign 1997